# Waste Face Surgical Mask Transformation into Crude Oil and Nanostructured Electrocatalysts for Fuel Cells and Electrolyzers

**DOI:** 10.1002/cssc.202102351

**Published:** 2021-12-09

**Authors:** Mohsin Muhyuddin, Jonathan Filippi, Luca Zoia, Simone Bonizzoni, Roberto Lorenzi, Enrico Berretti, Laura Capozzoli, Marco Bellini, Chiara Ferrara, Alessandro Lavacchi, Carlo Santoro

**Affiliations:** ^1^ Department of Materials Science University of Milano-Bicocca U5 Via Cozzi 55 20125 Milan Italy; ^2^ Istituto di Chimica Dei Composti OrganoMetallici (ICCOM) Consiglio Nazionale Delle Ricerche (CNR) Via Madonna Del Piano 10 50019 Sesto Fiorentino Firenze Italy; ^3^ Department of Earth and Environmental Sciences University of Milano-Bicocca Building U01 Piazza della Scienza 1 20126 Milan Italy

**Keywords:** crude oil, hydrogen evolution reaction, oxygen reduction reaction, surgical mask, valorization

## Abstract

A novel route for the valorization of waste into valuable products was developed. Surgical masks commonly used for COVID 19 protection by stopping aerosol and droplets have been widely used, and their disposal is critical and often not properly pursued. This work intended to transform surgical masks into platinum group metal‐free electrocatalysts for oxygen reduction reaction (ORR) and hydrogen evolution reaction (HER) as well as into crude oil. Surgical masks were subjected to controlled‐temperature and ‐atmosphere pyrolysis, and the produced char was then converted into electrocatalysts by functionalizing it with metal phthalocyanine of interest. The electrocatalytic performance characterization towards ORR and HER was carried out highlighting promising activity. At different temperatures, condensable oil fractions were acquired and thoroughly analyzed. Transformation of waste surgical masks into electrocatalysts and crude oil can open new routes for the conversion of waste into valuable products within the core of the circular economy.

## Graphical abstract

**Mask recycling**: Surgical masks are subjected to pyrolysis processes at controlled temperature and atmosphere in order to transform them into valuable products. Crude oil is formed during pyrolysis and analyzed. The char produced from the process is functionalized with metal‐phthalocyanine and then tested as the electrocatalyst for oxygen reduction reaction and hydrogen evolution reaction in neutral and alkaline media.

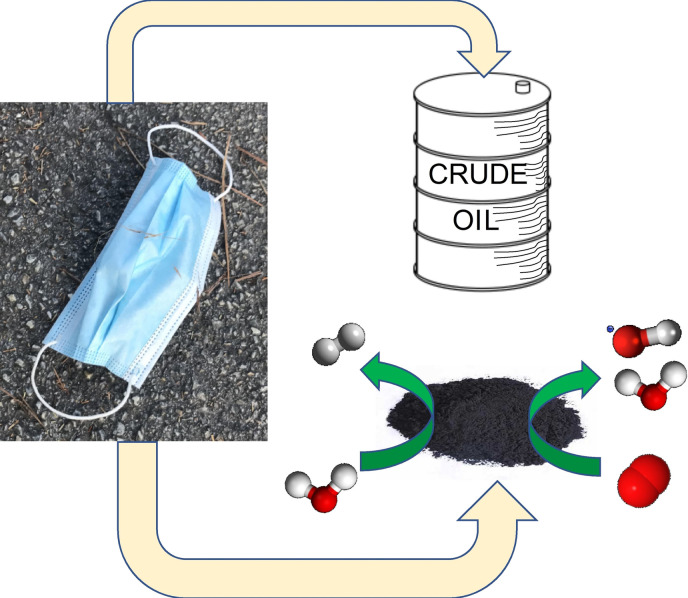

## Introduction

Since the end of 2019, the world is facing an unprecedented global pandemic due to a rapid spread of a contagious coronavirus disease known as COVID‐19, causing severe acute respiratory syndrome coronavirus 2 (SARS‐CoV‐2).[^1^, ^2^] This viral infection has gravely shaken human health, the world's economy, social interactions, and it has also aggravated the global plastic waste pollution due to inadequate disposal of personal protective equipment (PPE), causing enormous challenges to the ecological pyramid.[^3^, ^4^] Among the various viable strategies to quench the transmission of virus, surgical face masks are considered one of the fundamental defense‐line PPEs as the virus mainly diffuses through aerosol and droplets.[^5^, ^6^] Consequently, in parallel with the effective vaccination campaign, the use of face masks has become a must for everyone in the present scenario, which has sparked a massive demand for surgical face masks all over the world.

The most commonly used surgical face masks are typically composed of non‐woven polypropylene and/or polyethylene, which represent potential environmental risks upon their disposal due to their slow degradation often combined with a massive volume of overwhelming waste mismanagements.[^7^, ^8^] In addition to land pollution, a considerable proportion of face masks is erroneously discharged into oceanic bodies, polluting the aquatic mediums and marine environment.[^7^, ^9^, ^10^] Just for the year 2020, it was estimated that about 1.56 billion surgical face masks entered into oceanic bodies, which would be nearly equal to 4680–6240 metric tons of waste plastic.[^11^, ^12^] Such a huge volume of plastics and microplastics entering into the aquatic domains can potentially harm the marine creatures upon the ingestion or getting stuck into the plastic masks, which may either annihilate them in a direct way (i. e., impairment of reproduction and growth of young mammals) and/or render them more vulnerable to the other dangers.[^13^, ^14^, ^15^] In addition to marine life, waste face masks are also becoming the cause of premature deaths of fauna and flora due to choking and suffocating entanglement.^[9]^

The COVID‐19 PPE and masks cannot be recycled through the common plastic routes because in many countries they are considered a potential cause of biological hazard.[^16^, ^17^] Therefore, waste PPE are treated differently and are subject to sterilization, heat treatment, or other advanced disinfection processes that could deactivate or remove the virus. So far, incineration is a common route followed to reset the biological hazard and burn the plastic and, where it is possible, for recovering valuable heat and transforming it into electricity, but this methodology contradicts the technical merits of environmental pollution controls.^[18]^ However, as these PPE masks are mainly composed of plastic‐based materials, their valorization through transformation processes that include high‐temperature treatment steps (i. e. pyrolysis) reducing the biological hazard can be envisioned.^[19]^ So far, few studies have been presented on the transformation of PPE into valuable products within the core of the circular economy.

Recently, Dharmaraj et al. comprehensively summarized the scientific possibilities of waste COVID‐19 PPE handling, treatment, and generation of valuable energy products such as oil, gas, and char through pyrolysis processes of versatile nature.^[19]^ Jung et al. attempted to valorize disposable face masks into hydrogen and various ranges of important hydrocarbons via an environmentally benign pyrolysis process over a Ni/SiO_2_ catalyst.^[20]^ In another study, it was found that during the pyrolysis of disposable face masks non‐condensable hydrocarbons of great importance from a fuel point of view, such as CH_4_, C_2_H_4_, C_2_H_6_, C_3_H_6_ and C_3_H_8_, are evolved.^[11]^ Aragaw and Mekonnen launched a research to identify the polymer type of face masks and gloves and analyzed their transformation into crude oil via pyrolysis.^[21]^ Chen et al. investigated the thermodynamic characteristics of pyrolysis of waste medical surgical mask rope in the inert atmosphere and compared the obtained products with those of conventional plastics.^[22]^ Recently, Hu and Lin carbonized the waste polypropylene face masks into carbonaceous char, which was used as a cathode material for supercapacitor and provided promising specific capacitance of 328.9 F g^−1^ at a current density of 1 A g^−1^.^[23]^

In parallel to the disposal and valorization of surgical masks, the production of hydrogen and its utilization for electrical energy production is rising exponentially, mainly due to the push towards the replacement of fossil fuels with cleaner energy production technologies for fighting global warming. Several technologies including electrolyzers and fuel cells have even been deployed commercially. The main issue is the utilization of precious metals such as iridium and platinum, commonly employed in electrolyzers and fuel cells as electrocatalysts. These materials raise costs and hinder the large‐scale production and commercialization of the devices. Most electrolyzers and fuel cells operate in acidic environments, making it difficult to replace noble metals with noble metal‐free electrocatalysts. However, switching to neutral or even alkaline electrolytes might help significantly to keep high electrochemical performance and to utilize platinum group metal‐free (PGM‐free) electrocatalysts. In neutral media, technologies such as microbial fuel cells (MFC) and microbial electrolysis cells (MEC) are capable of producing electricity by degrading organics and producing electricity or producing hydrogen.[^24^, ^25^, ^26^] MFC and MEC rely on microorganisms acting as biocatalysts on the anode, while generally abiotic oxygen reduction reaction (ORR) and hydrogen evolution reaction (HER) occur on the abiotic cathode. Replacing platinum with PGM‐free alternatives is a must. In alkaline media, alkaline fuel cells oxidize hydrogen at the anode and reduce oxygen at the cathode.^[27]^ Alkaline electrolyzers instead evolve oxygen at the anode and evolve hydrogen at the cathode.[^28^, ^29^] For alkaline technologies, too, the replacement of expensive and rare electrocatalysts with more abundant and affordable materials is pursued.^[30]^

Great attention has been dedicated to the research and development of novel electrocatalysts that utilize earth‐abundant transition metals such as Mn, Fe, Co, Ni, and Cu with the main focus on the cathode reaction, which is usually the bottleneck of the systems. So far, metal–nitrogen–carbon (M−N−C) with metal as Mn, Fe, Co, Ni, Cu electrocatalysts for ORR[^31^, ^32^, ^33^, ^34^, ^35^, ^36^, ^37^, ^38^] and HER,[^39^, ^40^, ^41^, ^42^, ^43^, ^44^] respectively, have proven to be interesting and promising alternatives to platinum due to their electrocatalytic activities. These active sites are incorporated into a carbon matrix structure, which acts as conductive support.

To date, several studies have been conducted on the synthesis of high‐efficiency PGM‐free electrocatalysts derived from controlled pyrolysis processes of carbonaceous waste, showing a significant development in the paradigm of electrocatalysis.[^45^, ^46^, ^47^]

A novel synthesis pathway considers the utilization of waste organic compounds, mainly derived from waste biomass, as an excellent ingredient for the synthesis of electrocatalysts for specific electrochemical reactions. Recent reviews report the advancements done in this direction for the synthesis of ORR electrocatalysts.[^46^, ^48^, ^49^] Recently, also waste plastics were transformed into carbonaceous and PGM‐free electrocatalysts.[^45^, ^50^, ^51^, ^52^]

Interestingly, this route leads to the valorization of waste carbon‐containing sources that are transformed into conductive char, which is then functionalized with the appropriate metal–nitrogen moieties, resulting in an efficient electrocatalyst. These processes enhance the core of the circular economy approach where waste is transformed into a valuable product.

In this work, surgical masks are homogenized and pyrolyzed under controlled atmosphere and temperature, producing char and oil. The char is then characterized and activated using KOH and further functionalized through pyrolysis processes adding the phthalocyanine (Pc) of the metal of interest (FePc for ORR and NiPc for HER). The obtained electrocatalysts are tested in neutral and alkaline electrolytes for the ORR and HER. The oil samples obtained at 500, 700, and 900 °C are also characterized. This work successfully underlines a new possibility of recycling the waste surgical masks and transforming them into PGM‐free electrocatalysts for ORR and HER as well as recovering crude oil.

## Results and Discussion

### Pristine mask analysis

**Figure 1:**
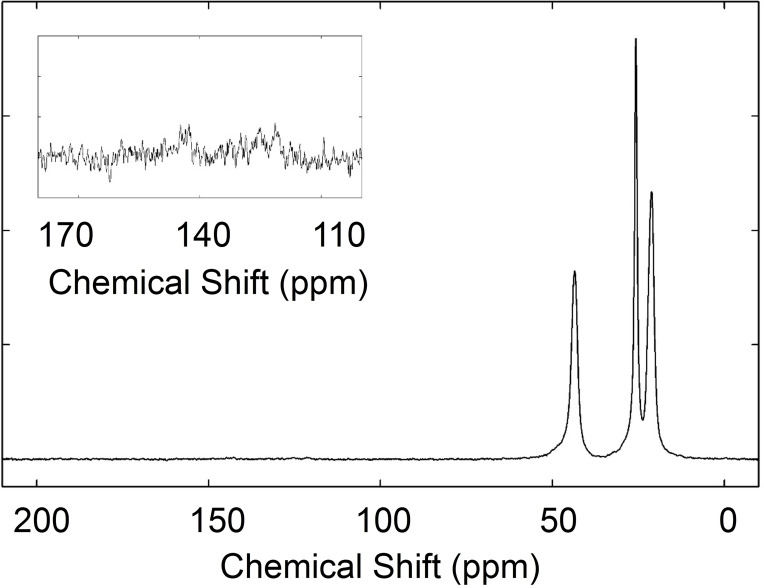
^13^C cross‐polarization magic angle spinning (CPMAS) (MAS 10 kHz) NMR spectrum of the pristine powders obtained by shredding of the PPE mask.

Solid‐state nuclear magnetic resonance (NMR) spectroscopy was used to identify the composition of the surgical masks used in this study (Figure 1). The spectrum is dominated by aliphatic signals (43.6, 25.7, 21.2 ppm and a small bump at 48.1 ppm), compatible with the polypropylene (PP) and polyethylene (PE) composition of the PPE mask[^22^, ^53^, ^54^, ^55^, ^56^] in agreement with the EU regulation (UNI EN 14683 : 2019+AC:2019). Very small signals are observed in the 160–110 ppm region and can be related to the presence of nylon‐6, compatible with infrared (IR) detection of polycarbonates. It must be mentioned that the nylon strips were removed from the surgical masks before pyrolysis; however, residual nylon, especially on the hot‐pressed mask‐elastic could have remained, explaining the small signal detected. The presence of such a small contribution is compatible with the composition of the PPE mask.

### Synthesis, morphology, and structure

After the structural and molecular investigation of pristine masks through NMR spectroscopy, they were subjected to atmosphere‐controlled (UTH N_2_) pyrolysis at 900 °C in order to obtain carbonaceous char (Mask‐Char), which afterward acted as a precursor for the development of carbon‐based PGM‐free electrocatalysts for ORR and HER. The initial step towards the fabrication of electrocatalysts was the activation of Mask‐Char with KOH to improve the surface area and to induce microporosity. The activation of carbon is generally considered due to the formation of K_2_CO_3_ at around 400 °C, which subsequently decomposes at 700 °C and provides supplementary pores in the carbon frameworks.^[57]^ In the next step, the “Activated Mask‐Char” was separately functionalized with FePc and NiPc via a systematic route to ensure molecularly dispersed M‐N_*x*_‐based active sites throughout the carbon matrix, and the samples formed were named as Mask‐Char‐Fe and Mask‐Char‐Ni, respectively.

It is known that Fe‐N_*x*_ active sites are primarily essential for improving the ORR activity,[^58^, ^59^, ^60^] whereas nickel coordinated with nitrogen (Ni‐N_*x*_) is generally believed to be an important influencer for HER electrocatalysis.[^39^, ^61^] Traditionally M−N−C type of electrocatalysts are synthesized via thermochemical conversion at an elevated temperature using precursors containing the metal and a nitrogen source such as inorganic metal salts and nitrogen sources, metal–organic framework (MOF), covalent organic framework (COF), or others. However, the incorporation of a nitrogen source into carbon generally gives rise to occlusion of primary pores of the carbon support due to which the original textural properties of the carbon are considerably affected.[^62^, ^63^] Recently, Mazzucato and Durante established the fact that efficacious Fe‐N_*x*_ can only be generated at numerous accessible locations when the ligands comprising of aromatic‐N rings are used because during their decomposition microporosity is increased, which enhances the overall ORR activity.^[62]^ After reviewing the utilization of various metal‐N_4_ chelate macrocycles for ORR, de Oliveira et al. reported the synthesis of FePc supported over different carbons matrixes.^[64]^ FePc is not only an attractive iron source owing to its exclusive characteristics of centrosymmetric arrangement, the ability to donate electrons, bulky conjugated molecular structure, and robust interactions among the aromatic rings, but also it is a concurrent source of nitrogen.[^64^, ^65^, ^66^]

Similarly, NiPc proved to be an efficient and robust catalyst for the evolution of hydrogen due to its stable Ni−N sigma bond.^[67]^ However, the direct application of metallic phthalocyanine for ORR electrocatalysis could lead to inferior electronic conductivity, short‐term stability, production of peroxide during ORR, inhomogeneous dispersion of active sites, aggregation of nanoparticles, and formation of the condensed microstructure with a higher degree of graphitization due to the catalytic effect of the central metal.[^62^, ^64^, ^68^, ^69^] Consequently, to overcome such shortcoming conductive carbon support is obligatory to enhance the electrocatalytic activity using high temperature processes. For these reasons, waste surgical masks were subjected to pyrolysis (Mask‐Char), then activated with KOH (Activated Mask‐Char), and finally functionalized with Fe (Mask‐Char‐Fe) or Ni (Mask‐Char‐Ni). The metal‐containing catalysts were washed with acid after the pyrolysis and then subjected to another pyrolysis in a slightly reducing atmosphere. The four samples prepared during this study are summarized in Table 1.

**Table 1:** Designation of the acronyms used for the identification of the developed electrocatalysts.

Acronym	1st pyrolysis	Activation	2nd pyrolysis	Acid washing	3rd pyrolysis
Mask‐Char	900 °C in N_2_	–	–	–	–
Activated Mask‐Char	900 °C in N_2_	KOH	–	–	–
Mask‐Char‐Fe	900 °C in N_2_	KOH	FePc 900 °C in N_2_	0.5 m H_2_SO_4_	FePc 900 °C in N_2_/H_2_
Mask‐Char‐Ni	900 °C in N_2_	KOH	NiPc 900 °C in N_2_	0.5 m H_2_SO_4_	NiPc 900 °C in N_2_/H_2_

**Figure 2:**
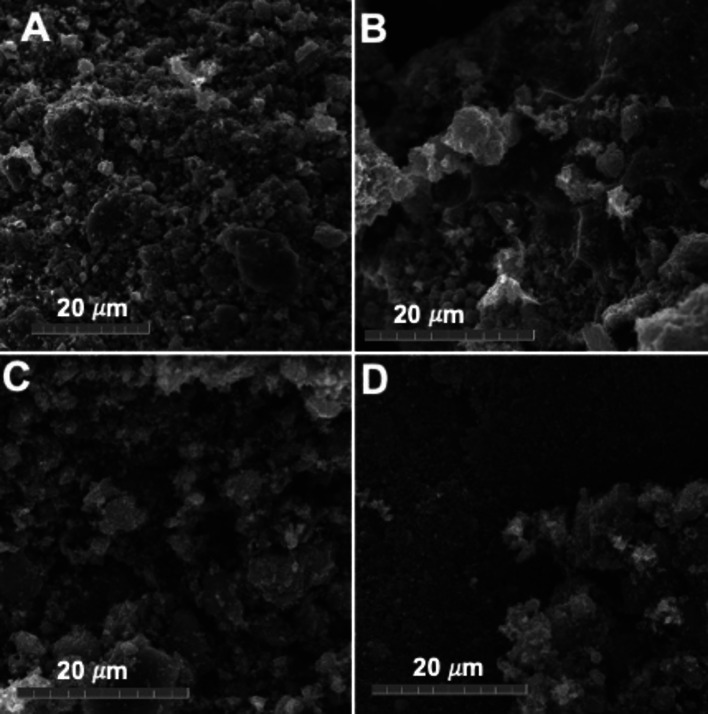
SEM images of (A) Mask‐Char, (B) Activated Mask‐Char, (C) Mask‐Char‐Fe, and (D) Mask‐Char‐Ni.

The surface of the electrocatalysts obtained was characterized by several techniques to study the morphological features and the surface chemistry. Scanning electron microscopy (SEM) images were taken of the Mask‐Char (Figure 2A), activated Mask‐Char (Figure 2B), Mask‐Char‐Fe (Figure 2C) Mask‐Char‐Ni (Figure 2D). The formation of additional pores on the surface after the activation process is visible. Moreover, a visual increase in the roughness of the surface can be seen after activation. However, despite giving some qualitative information related to the surface, conclusive info simply using SEM cannot be obtained. Brunauer–Emmett–Teller (BET) surface area and Barrett–Joyner–Halenda (BJH) average pore size were investigated for the four samples to identify the variation of the BET surface area and the BJH average pore size with the functionalization steps. The results are shown in Table 2.

**Table 2:** BET surface area analysis and BJH average pore size of the Mask‐Char, Activated Mask‐Char, Mask‐Char‐Fe, and Mask‐Char‐Ni.

Sample	BET surface area [m^2^ g^−1^]	BJH desorption average pore size [nm]
Mask Char	31.9	36.4
Activated Mask‐Char	396.0	5.1
Mask‐Char‐Fe	296.5	14.9
Mask‐Char‐Ni	309.7	15.5

BET surface area increased from 31.9 (Mask Char) to 396.0 m^2^ g^−1^ (Activated Mask Char) after the activation with KOH (Table 2). The activation with KOH was intentionally performed in order to increase the surface area. BET surface area decreased by 25 % after the second pyrolysis, acid washing, and third pyrolysis (Table 2). In fact, the BET surface area of Mask‐Char‐Fe was 296.5 m^2^ g^−1^, and that of Mask‐Char‐Ni was 309.7 m^2^ g^−1^ (Table 2). This decrease was probably due to the addition of metal‐phthalocyanine that can lead to some agglomeration within the activated Mask‐Char. The samples’ average pore diameter spans from 5.1 nm for Activated Mask‐Char to 36.4 nm of Mask Char, falling in the range of mesoporous materials (Table 2). Importantly, the narrower value was measured after activation and increased to the range of 14.9–15.5 nm after the functionalization with Fe‐ and Ni‐phthalocyanine. This analysis was conclusive in order to underline that the process of activation led to a desired increase in the BET surface area.

Energy‐dispersive X‐ray spectroscopy (EDS) was carried out from the surface of char, activated char, and functionalized char as reported in the Supporting Information (Figures S1–S4). The majority of the samples were composed of carbon and oxygen. Traces of Na, Mg, K, S, Cl, and Ca were found indicating a possible anthropic contamination during the production process. The presence of Cr could be attributed to the residual stainless‐steel nose‐holder that was removed from the surgical mask before pyrolysis, while the presence of Al can be due to the contamination with the crucible used during pyrolysis. It might be possible that part of the crucible was scraped out when recovering the char sample after pyrolysis. Importantly, Ti was found at atomic weight percentage above 1 % in the analyzed samples. This can be associated due to the utilization of TiO_2_ for bleaching the internal part of the surgical mask, the color of which is typically whitish compared to the external part, which is light blue. The electrocatalysts synthesized through functionalization of FePc and NiPc showed small peaks of Fe and Ni in their EDS spectra.

**Figure 3:**
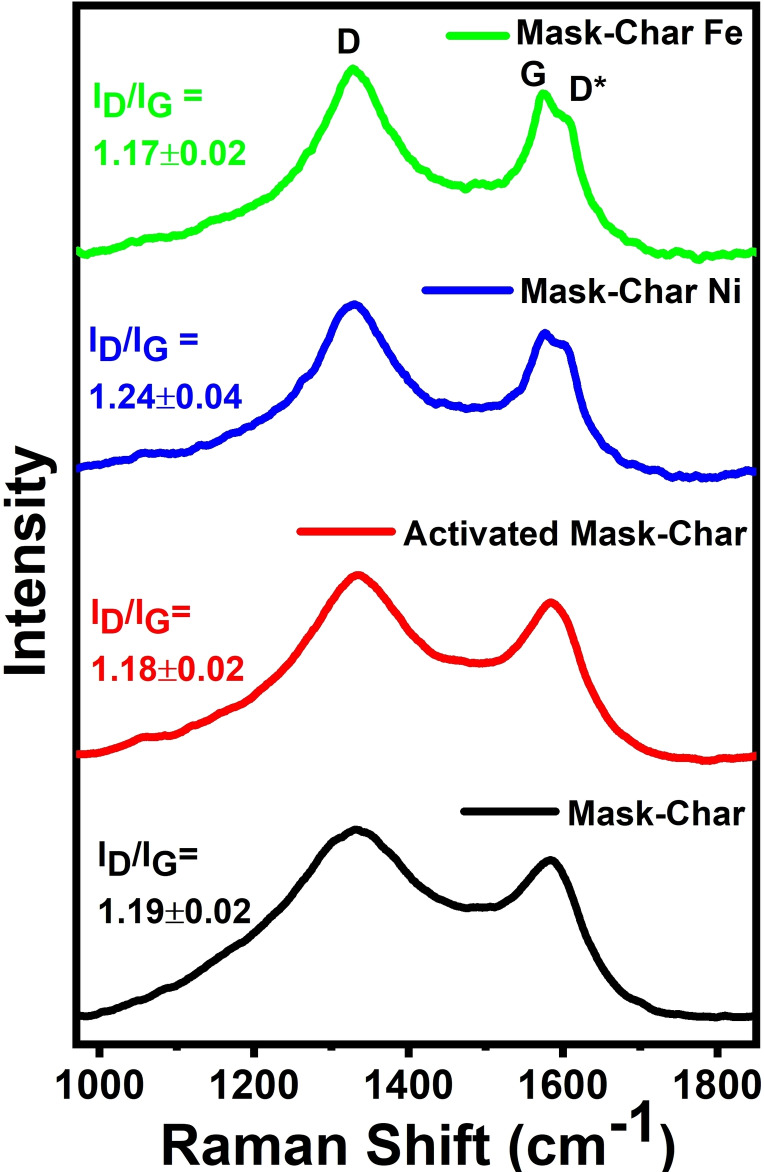
Representative Raman spectra for Mask‐Char (black), Activated Mask‐Char (red), Mask‐Char‐Ni ( blue), and Mask‐Char‐Fe (green)

Raman spectroscopy serves as one of the fundamental non‐destructive techniques to investigate the corresponding amorphous and graphitic contents of nanoscale carbons. Therefore, all the carbonaceous powdered samples derived from masks were analyzed with Raman spectroscopy, and the resultant spectra are illustrated in Figure 3. Each sample exhibited both the *D* band and *G* band, which correspond to *K*‐point phonons of A_1g_ symmetry with breathing mode of highly disordered sites and zone center phonons of E_2g_ symmetry with a translational motion of ideal sp^2^‐type graphitic lattices, respectively.^[70]^ The G band is a characteristic feature of the ordered graphitic structure, whereas the D band is forbidden in perfect graphitic structures and typically originates from broken edges, disruption of graphitic structure, and reduction of *a*‐axis coherence lengths (*L*_a_).^[71]^ Thus, the intensity ratio of D to G band (*I*_D_ */I*_G_) is a common indicator of defect density. From Figure 3, it can be appreciated that after KOH activation the D peak becomes broader, while *I*_D_ */I*_G_ remains constant. The maximum ratio of *I*_D_ */I*_G_ of 1.24 was achieved in the case of Mask‐Char‐Ni, whereas in the case of iron doping the *I*_D_ */I*_G_ ratio was moderately quenched to 1.17. Daniel et al., while preparing Fe−N−C ORR electrocatalysts from waste polyurethane, also noticed an improvement in the degree of graphitization during pyrolysis mainly due to iron.^[45]^ On the other hand, Mask‐Char‐Ni also exhibits a shoulder on the left side of D band, which could possibly be originated by the presence of a diamond‐like sp^3^‐rich phase.^[72]^ In addition to the typical D and G band, the doped samples (Mask‐Char‐Ni and Mask‐Char‐Fe) also exhibited an extra Raman feature in the vicinity of 1605 cm^−1^ (D* band) on the right‐hand shoulder of the G band, which is also attributed to structural defects.^[73]^ The D* band characteristically emerges due to intra‐valley double resonance scattering, in which the defect‐related discontinuities offer the lost momentum needed to satisfy the resonant process.[^74^, ^75^, ^76^, ^77^] The comparative strength of the D* band specifies the occurrence of a large number of defects; either present as in‐plane heteroatom substitution, atomic vacancies, and/or destructive grain boundaries and edges.

### Electrochemical results

After activation and functionalization, the obtained electrocatalysts were tested for ORR and HER at two different operational pH values: neutral pH using 1 m phosphate buffer solution (PBS) and alkaline conditions using 0.1 m potassium hydroxide (KOH) electrolyte. Particularly for ORR, the samples Mask‐Char, Activated Mask‐Char, Mask‐Char‐Ni, and Mask‐Char‐Fe were tested and compared. For HER instead, Mask‐Char, Activated Mask‐Char, and Mask‐Char‐Ni were studied.

#### Oxygen reduction reaction in neutral media

**Figure 4:**
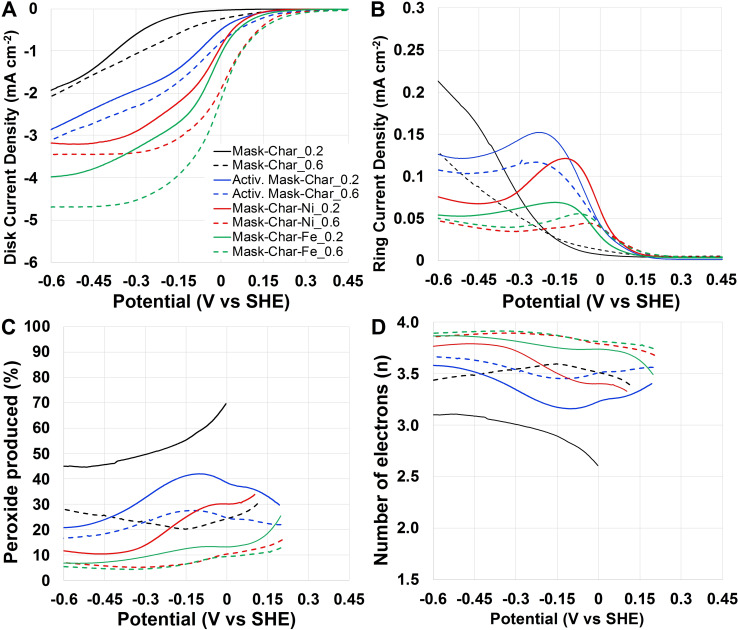
(A) Disk current, (B) ring current, (C) peroxide produced, (D) number of electrons transferred for Mask‐Char (black), Activated Mask‐Char (blue), Mask‐Char‐Ni (red), and Mask‐Char‐Fe (green) in saturated oxygen neutral electrolyte (1 m PBS).

Mask‐Char‐Fe and Mask‐Char‐Ni electrocatalysts derived from pyrolyzed and functionalized surgical masks were tested for ORR and compared to Mask‐Char and Activated Mask‐Char in neutral media (Figure 4). The disk current density recorded during the linear sweep voltammetry (LSV) of the developed electrocatalysts run at 1600 rpm operating in neutral media are plotted considering the potential vs. standard hydrogen electrode (SHE) and shown in Figure 4A. In neutral media, the onset potential (*E*_on_) was approximately 0.25 and 0.35 V vs. SHE for Mask‐Char‐Fe and Mask‐Char‐Ni at catalyst loadings of 0.2 and 0.6 mg cm^−2^, respectively. For Activated Mask‐Char, *E*_on_ was approximately 0.25 and 0.3 V vs. SHE (loadings of 0.2 and 0.6 mg cm^−2^). Lowest *E*_on_ of approximately 0.0 (loading 0.2 mg cm^−2^) and 0.2 V vs. SHE (loading 0.6 mg cm^−2^) was detected for Mask‐Char. Interestingly, a different *E*_on_ was detected at different loadings, and this is not obvious since *E*_on_ is an intensive parameter and should be independent of the catalyst loading. Importantly, it can be noticed that the activation process enhanced the current produced, and this might be related to the increase in the surface area measured through BET analysis (Figure 4A). Further improvement was noticed with the functionalization with Fe and Ni (Figure 4A). Each step in the synthetic process has led to an improvement in electrocatalytic activity.^[78]^ The shape of the LSV sigmoids, in fact, changed with the presence of Ni and Fe into the electrocatalyst with important improvements (a shift toward lower overpotentials) in the half‐wave potential (*E*_1/2_) (Figure 4A). In all the cases, an increase in the catalyst loading from 0.2 to 0.6 mg cm^−2^ led to an improvement in the current produced in agreement with previously reported works operating in neutral media.[^79^, ^80^, ^81^] *E*_1/2_ was approximately 0 V vs. SHE for Mask‐Char‐Fe and Mask‐Char‐Ni (loading 0.6 mg cm^−2^) and roughly 50 mV lower for lower catalyst loading. The limiting current densities for Mask‐Char‐Fe were higher than the one produced by Mask‐Char‐Ni (Figure 4A). In any case, the overpotentials were high, indicating a low electrocatalytic activity, which is typical in neutral media due to the low concentration of H^+^ and OH^−^ within the electrolyte. The ring current measured during the LSV is reported in Figure 4B. It must be underlined that the ring current density decreased with the increase in the loading (Figure 4B). Interestingly, for Mask‐Char, the ring current increased with the potential decrease. In parallel, for Activated Mask‐Char, Mask‐Char‐Ni, and Mask‐Char‐Fe, the peroxide increased at lower overpotentials and reached a plateau. This might lead to the speculation that Mask‐Char was a peroxide‐producing electrocatalyst while Activated Mask‐Char, Mask‐Char‐Ni, and Mask‐Char‐Fe were peroxide‐producing electrocatalysts at lower overpotentials and peroxide‐scavenging electrocatalysts at higher overpotentials. The percentage of peroxide was measured during LSV and reported in Figure 4C. Generally, it can be noticed that the peroxide produced decreased moving from Mask‐Char to Activated Mask‐Char and finally to the electrocatalyst functionalized with Fe or Ni. For Mask‐Char‐Ni (loading of 0.2 mg cm^−2^), the peroxide detected varied between 30 % (0.1 V vs. SHE) and 10 % (−0.5 V vs. SHE). For Mask‐Char‐Fe (loading 0.2 mg cm^−2^), the peroxide produced varied between 25 % (0.1 V vs. SHE) and 7 % (−0.5 V vs. SHE). For loading of 0.6 mg cm^−2^, the peroxide detected was lower and particularly for Mask‐Char‐Ni and Mask‐Char‐Fe varied between 13–15 % (0.2 V vs. SHE) and 7–8 %. Importantly, an increase in the catalyst loading led to a decrease in the peroxide produced, indicating that the peroxide is reduced to the final product within the thick electrocatalyst layer.[^79^, ^82^] The number of electrons exchanged for Mask‐Char‐Fe and Mask‐Char‐Ni was higher than Activated Mask‐Char and Mask‐Char (Figure 4D). Particularly, Mask‐Char‐Fe and Mask‐Char‐Ni had a number of electrons exchanged around 3.8–3.9. Activated Mask‐Char had a maximum electron transfer of 3.7 (0.6 mg cm^−2^ catalyst loading) that was slightly higher compared to Mask‐Char (3.4–3.6).

#### Oxygen reduction reaction in alkaline media

**Figure 5:**
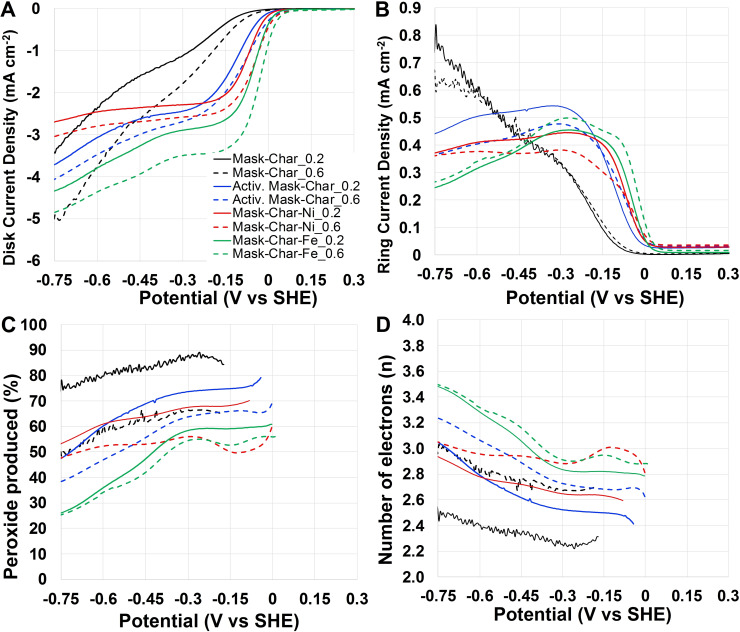
(A) Disk current, (B) ring current, (C) peroxide produced, (D) number of electrons transferred for Mask‐Char (black), Activated Mask‐Char (blue), Mask‐Char‐Ni (red), and Mask‐Char‐Fe (green) in saturated oxygen alkaline electrolyte.

The trend of disk current densities from LSV of the electrocatalysts using alkaline electrolyte is reported in Figure 5A. In alkaline media instead, *E*_on_ was around 0.0 (Mask‐Char) and 0.05–0.1 V vs. SHE (Activated Mask‐Char, Mask‐Char‐Ni, and Mask‐Char‐Fe). At catalyst loading of 0.2 mg cm^−2^, _1/2_) was approximately −0.05, 0.07, and 0.12 V vs. SHE for Mask‐Char‐Fe, Mask‐Char‐Ni, and Activated Mask‐Char, respectively. For higher loading (0.6 mg cm^−2^), *E*_1/2_ was slightly higher, measuring around −0.02 (Mask‐Char‐Fe), −0.05 (Mask‐Char‐Ni), and −0.085 V vs. SHE (Activated Mask‐Char). It can be noticed that the activation process during the synthesis improved the current produced as well as the *E*_1/2_. An important improvement in the sigmoid was detected after the functionalization with iron and nickel, with the first outperforming the second. The improvement achieved during the synthetic process was presented through microscopic and spectroscopic techniques^[83]^ and more recently using synchrotron spectroscopy.^[84]^

The increase in catalyst loading on the disk led to an increase in the disk current density (Figure 5A). In Figure 5B, the ring current measured during the LSV is reported. Similar to neutral media, Mask‐Char again demonstrated an increased ring current density with the decrease in potential in alkaline media. Activated Mask‐Char, Mask‐Char‐Ni, and Mask‐Char‐Fe had higher ring current density at lower overpotentials that decreased with higher overpotentials. The peroxide produced was higher compared to neutral media (Figure 5C). The electrocatalyst producing lower peroxide was the one containing iron (Mask‐Char‐Fe) that had over 60 % peroxide produced at low overpotentials (0 V vs. SHE) down to roughly 25 % at high overpotentials (−0.75 V vs. SHE). The functionalization with iron contributed to the presence of active sites following a direct 4‐electron transfer mechanism and also enhanced the peroxide scavenging. Higher peroxide was detected for Mask‐Char and Activated Mask‐Char. The number of electrons exchanged was also calculated and presented in Figure 5D. For ORR, a number of electrons close to 4 is preferred, indicating an efficient ORR and low production of intermediates that could be deleterious for long‐term operation in fuel cells. It can be seen that the maximum number of electrons increased for Mask‐Char, Activated Mask‐Char, Mask‐Char‐Ni, and Mask‐Char‐Fe, respectively (Figure 5D). The number of electrons exchanged underlined the improvement achieved after the functionalization with the iron‐phthalocyanine and the formation of metal‐N_*x*_‐C active sites. Different electron transfer mechanisms occurring on the same electrocatalysts at different pH values were described in detail in recent literature, underlining the motives for higher peroxide produced in alkaline media.^[85]^

#### ORR Tafel analysis

**Figure 6:**
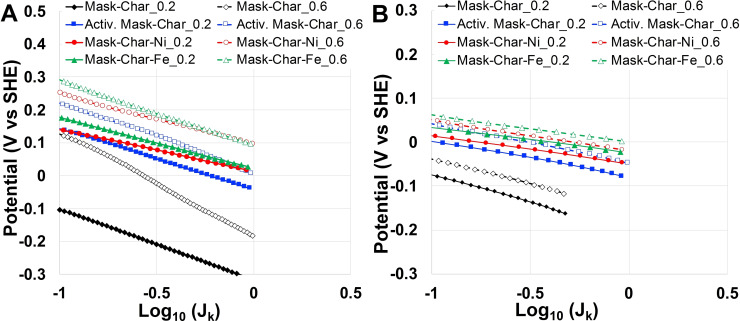
Tafel plots for ORR regarding the electrocatalysts studied in (A) neutral and (B) alkaline media.

ORR Tafel analysis on the electrocatalysts objective of this study was conducted, and the Tafel plots are shown in Figure 6. The Tafel slopes are summarized in Table 3. The Tafel slopes for ORR between 128 and 195 mV dec^−1^ indicate slow kinetics in neutral media. Surprisingly, increasing the loading slowed the kinetics to some extent, probably introducing some form of aggregation of the particles, or introducing mass transport problems through the catalytic layer even at currents much lower than the convection limited current. This behavior is also present and more severe in the pristine materials (i. e., without deposition and pyrolysis of phthalocyanines). Regarding Tafel analysis in alkaline media, the electrocatalysts show a Tafel slope for ORR between 58 and 69 mV dec^−1^. While increasing the loading did not affect the kinetics appreciably, the kinetics are significantly faster than in neutral media. These Tafel slopes are similar to the one reported for Pt^[86]^ and other PGM‐free electrocatalysts.^[87]^

**Table 3:** ORR Tafel slopes for the electrocatalysts operating in neutral and alkaline media.

Sample	catalyst loading [mg cm^−2^]	Tafel slope [mV dec^−1^]	
		neutral	alkaline
Mask Char	**0.2**	215	128
Mask Char	**0.6**	323	116
Activated Mask‐Char	**0.2**	186	82
Activated Mask‐Char	**0.6**	214	87
Mask‐Char‐Ni	**0.2**	128	63
Mask‐Char‐Ni	**0.6**	152	69
Mask‐Char‐Fe	**0.2**	157	58
Mask‐Char‐Fe	**0.6**	195	61

#### HER in neutral and alkaline media

**Figure 7:**
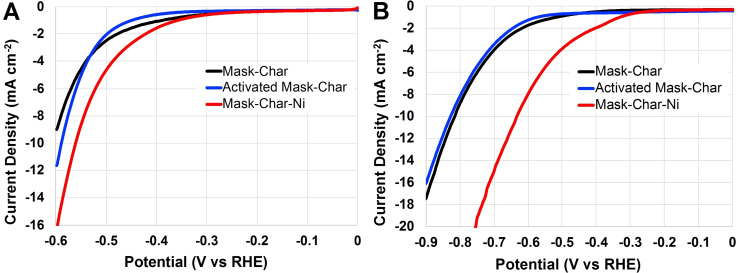
LSVs in saturated nitrogen electrolyte for Mask‐Char, Activated Mask‐Char, and Mask‐Char‐Ni. (A) Test done in neutral media. (B) Experiments conducted in alkaline electrolyte.

Char from surgical masks was also functionalized for HER by adding nickel functionalities. Particularly, nickel phthalocyanine was homogenized with the activated char and then subjected to controlled pyrolysis, acid washing, second pyrolysis, and then ball milling. The obtained catalysts possessing active sites of the type Ni‐N_*x*_‐C were tested for HER. Particularly, LSVs from 0 to −0.6 V vs. reversible hydrogen electrode (RHE) were run using neutral electrolytes, and LSVs from 0 to −0.9 mV vs. RHE were run in alkaline electrolytes. The LSVs were run at 1600 rpm and were plotted vs. RHE in neutral (Figure 7A) and alkaline media (Figure 7B). The parameters of interest were *E*_on_ and the overpotentials at current density of 10 mA cm^−2^. Concerning neutral media (Figure 7A), *E*_on_ was approximately −0.25 V vs. RHE for Mask‐Char‐Ni and Activated Mask‐Char. Lower *E*_on_, approximately‐0.35 V vs. RHE, was obtained for Mask‐Char. Interestingly, Activated Mask‐Char generated more current at higher overpotentials compared to Mask‐Char electrocatalyst. In particular, at the current density of 10 mA cm^−2^, Mask‐Char‐Ni had an overpotential of around 0.55 V. Importantly, Mask‐Char‐Ni always overperformed the other two samples. In alkaline media (Figure 7B), *E*_on_ was around −0.3 V vs. RHE for Mask‐Char‐Ni and around −0.4 V vs. RHE for Mask‐Char and Activated Mask‐Char. The overpotentials at a current density of 10 mA cm^−2^ were approximately 0.62 V for Mask‐Char‐Ni and 0.82 V for Mask‐Char and Activated Mask‐Char. Importantly, in alkaline media, the functionalization with Nickel led to a decrease of the overpotentials of approximately 0.2 V, which is certainly an improvement but still, the overpotentials remained quite high.

### Crude oil derived from pyrolysis

**Figure 8:**
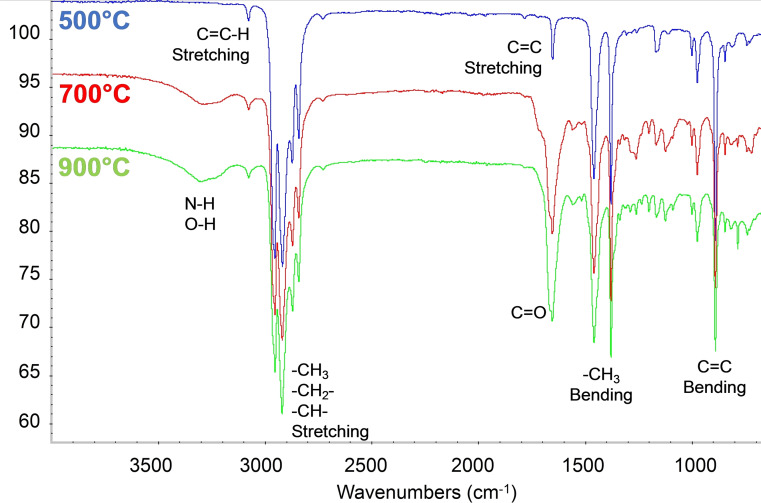
FTIR spectra of the condensable fractions from surgical mask pyrolysis at different temperatures (500, 700, and 900 °C).

In order to characterize the pyrolysis products, condensable fractions evolved from surgical mask pyrolysis were collected through a cold solvent trap and analyzed by different techniques. Fourier‐transform (FT)IR spectroscopy is a suitable technique to preliminarily investigate and characterize the chemical structures of complex materials aroused by a pyrolytic process of heterogeneous materials. In fact, its sensitivity towards terminal C=C and C=O bonds, generated during the pyrolysis, allows the thermal degradation progress to be measured.^[88]^ In Figure 8, the FTIR spectra of the condensable fractions from surgical mask pyrolysis at different temperatures (500, 700, and 900 °C) were reported. All spectra were similar to each other, displaying the characteristic peaks of polypropylene (PP),[^89^, ^90^, ^91^] the main polymer used in surgical mask: 973 and 996 cm^−1^ (rocking −CH_3_), 1166 cm^−1^ (wagging C−H), 1376 and 1456 cm^−1^ (symmetrical bending −CH_3_), and 2840, 2870, 2920, and 2950 cm^−1^ (stretching C−H). New bands were detected at 3050, 1650, and 887 cm^−1^ that are owing to the double bond functionality (stretching C=C−H, stretching C=C, bending C=C, respectively). The double bond functionalities were likely generated by different radical reactions on the PP chains followed by a β‐scission.^[92]^ In particular, by monitoring the 1650 and 887 cm^−1^ bands, the degradation of the polymer could be followed, but no significant differences in the absorbance of those bands were highlighted on the spectra at the investigated temperature. It is noteworthy that no further distinction between different types of double bonds can be made by IR spectroscopy. Nevertheless, FTIR spectroscopy was useful to evaluate the absence of linear olefin (by the absorbance ratio of the bands at 1376 and 1456 cm^−1^) and the isotacticity (by the absorbance ratio of the bands at 996 and 973 cm^−1^)^[93]^ compared with a reference spectrum of isotactic PP. The main differences were observed at 3300 and 1660 cm^−1^: at 700 and 900 °C, new bands were detected and related to the presence of N−H, O−H, and carbonyl C=O bonds, compatible with the presence of nylon‐6, as already evidenced with solid‐state NMR spectroscopy. It is possible that at higher pyrolysis temperature the nylon‐6 of the surgical mask started to degrade on the ϵ‐caprolactame monomer^[94]^ and/or partial chain oxidations with insertion of hydroxy groups took place.

**Figure 9:**
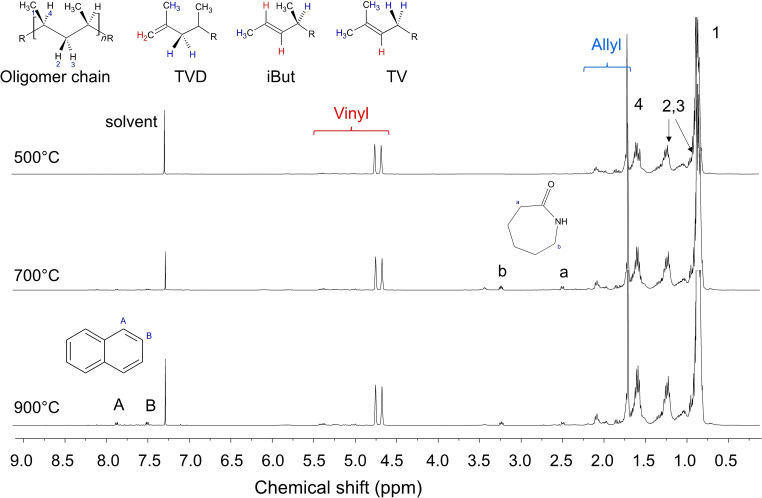
^1^H NMR spectra of the condensable fractions from surgical mask pyrolysis at different temperatures (500, 700, and 900 °C).

More and complementary information could be obtained by ^1^H NMR spectroscopy. In Figure 9, ^1^H NMR spectra of the condensable fractions from surgical mask pyrolysis at different temperatures (500, 700, and 900 °C) were reported.

The NMR spectra were similar and dominated by the presence of PP degradation products. In particular, the multiplet peaks at 0.80, 1.25, and 1.70 ppm (1–4) were easily ascribed to methyl, methylene, and methine H of the PP oligomeric chains. From the chemical shift of methylene H (2, 3) it was also possible to confirm a predominant isotacticity of the chains as detect by FTIR spectroscopy. Moreover, peaks in the 1.75–2.25 and 4.5–5.5 ppm ranges were also detected and related to the presence of double bonds. The multiplets at 1.75–2.25 ppm were related to the allyl H, and those at 4.5–5.5 ppm to the vinyl H. More specifically, at 1.75–2.25 ppm different H signals (highlighted in blue color; chemical structures reported in Figure 9) were detected and related to the TVD (terminal vinylidene), iBut (terminal isobuthylene), and TV (terminal vinylene) units. At 4.5–5.5 ppm it was possible to detect the corresponding vinyl hydrogen of those units, highlighted in red color. From the spectra, it is possible to observe that the terminal vinylidene units (1.70, 4.45, and 4.55 ppm) were the most common terminal functionality of the condensable products. For the samples pyrolyzed at higher temperature (700 and 900 °C), peaks related to ϵ‐caprolactame (multiplet at 2.5 and 3.25 ppm) and naphthalene (7.5 and 7.8 ppm) were detected,^[95]^ confirming the increase of signal in FTIR spectra of carbonyl bonds (caprolactame). Small signals in the 3–4 ppm region at 700 and 900 °C were detected, which may arise from alcohols confirming the 3300 cm^−1^ bands from FTIR spectra. ^1^H NMR spectroscopy was also suitable to obtain the number‐average molecular weight (*M*_n_) of the samples at advanced pyrolysis stages by measuring the relative content of end chain groups.[^88^, ^89^, ^96^] Consequently, the molecular weight can be expressed in terms of f_t_ (terminal functionalities) as *M*_n_=([CH_3_]/[TVD+TV+iBut])×f_t_×42 [g mol^−1^] where [CH_3_] is the whole methyl content. As far as the molar mass estimation from the ^1^H NMR spectra is concerned, the whole end‐capping double bond content (TVD at 4.7 and 4.8 ppm; iBut at 4.9 and 4.5 ppm; TV at 5.5 and 5.3 ppm) is taken against the total monomer one, as it is measured by the methine signal (the multiplet centered at 1.65 ppm). In this case, f_t_ is assumed to be 2 for the pyrolyzed samples.^[88]^ As results the *M*_n_ were estimated and reported in Table 4.

**Table 4:** *M*_n_ and chain length (C_*x*_) of the condensable fractions from surgical mask pyrolysis at different temperature (500, 700, and 900 °C) estimated by ^1^H NMR spectroscopy.

Pyrolysis temperature [°C]	*M*_n_ [g mol^−1^]
500	190 (C_14_)
700	208 (C_15_)
900	238 (C_17_)

**Figure 10:**
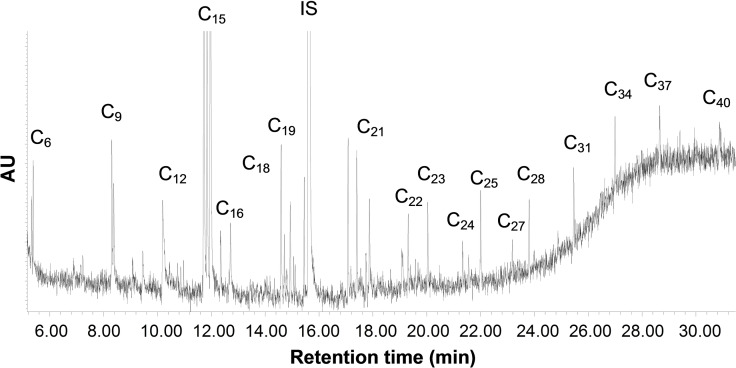
GC–MS chromatograms of the condensable fractions from surgical mask pyrolysis at 700 °C.

The samples were finally submitted to GC–MS characterization. Based on the mass spectra and literature accounts,^[20]^ a tentative attribution of the different chromatographic peaks was performed and reported in Figure 10 for the 700 °C sample, considered as the more representative. The condensable products from pyrolysis of surgical musk were composed prevalently by hydrocarbons form C_6_ and C_40_. Traces of aromatic compounds such as naphthalene and indene were detected only at 700 and 900 °C (confirming the ^1^H NMR spectroscopy observation).

**Figure 11:**
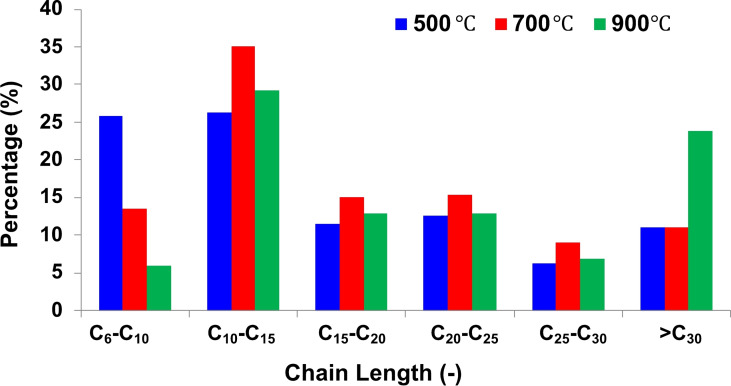
Quantification [%] from GC chromatogram of different range of chain length.

The hydrocarbons identified in the pyrolytic condensable fraction were classified based on the length of the oligomeric chain in C_6_−C_10_, C_10_−C_15_, C_15_−C_20_, C_20_−C_25_, C_25_−C_30_, and >C_30_, and, from the chromatographic integration, a percentage quantification was performed and reported in Figure 11.

In Figure 11, the fraction percentage of different range of chain length identified at the three different temperatures are reported. These fractions were calculated on the basis of the chromatographic peak area of each molecular weight range with respect to the total area of peaks. The sums are, respectively, 94, 99, and 92 % at 500, 700, and 900 °C due to the presence of small chromatographic peaks not attributed to aliphatic compounds. The yield of the lighter fraction (C_6_−C_10_) was higher at 500 °C and decreased at 700 and 900 °C (Figure 11). The main fraction recovered was the C_10_−C_15_ for all the samples, while the trend for the heaviest fraction (>C_30_) was opposite with the higher content for the 900 °C sample. Those results agree with the *M*_n_ estimation based on ^1^H NMR spectroscopy (Table 4), which indicated an increase of the average molecular weight of the condensable products along with the increase of pyrolysis temperature. This is probably because an increase in the number of carbons makes intermolecular attractive forces cumulatively more considerable, resulting in high boiling points, as observed by Park et al.^[11]^

### Potential benefits and outlook

Very large quantities of surgical masks are used on daily basis for the protection against COVID‐19. As mentioned in the introduction, the disposal of these medical devices is quite challenging, and often the wrong disposal ends with the release of plastic‐based masks into soil and water bodies, compromising the environmental ecology. To diminish the biological hazard of surgical masks, a heating treatment is required. The straightforward technique for handling waste surgical masks is the incineration and the possible recovery of waste heat for the production of electricity or heating water. However, other routes have been pursued by valorizing waste and transforming it into a valuable product within the core of the circular economy. Surgical masks are mostly composed of plastic materials that could be transformed into value‐added products (VAPs). Particularly, surgical masks have been transformed into crude oil, biochar, and other plastic materials.[^11^, ^17^, ^19^, ^20^, ^21^, ^23^] In this work, surgical masks have been characterized through solid‐state NMR spectroscopy to identify the composition, which is predominantly PP. Surgical masks were subject to controlled temperature and atmosphere pyrolysis, and their products were analyzed. During pyrolysis, crude oil was produced, and its composition at 500, 700, and 900 °C was analyzed with a larger quantity being C_10_−C_15_. The produced char was then functionalized to form metal‐N_*x*_−C centers on the conductive char. Particularly, the electrocatalyst used for ORR contained Fe to biomimic the ORR occurring in nature. The electrocatalyst used for HER instead contained Ni. The electrochemical results were promising, but still, improvements are required to meet the requirements of technology. This work shows an important possibility of valorizing surgical masks into valuable PGM‐free electrocatalysts for the hydrogen production and conversion technologies. This novel route might be implemented at a large scale relieving the problem of disposal of surgical masks. Nevertheless, some scientifically rational modifications in the adopted pathway could be made to further increase the yield and quality of carbonaceous char from the pyrolysis of waste masks and other medical PPE, which are otherwise far away from the common recycling routes Certainly, the electrochemical performance obtained is still limited compared to state‐of‐the‐art PGM electrocatalyst; however, improvements can be achieved by varying the pyrolysis processes such as temperature, atmosphere, time of pyrolysis, and type and quantity of the precursor. Functionalization with various low‐cost and readily available precursor ligands and inorganic metallic salts could also be a part of prospective research endeavors. Deeper attention should be devoted to the formation of the porosity and development of the active sites on the carbonaceous backbone. This initial work might open new routes for transforming surgical masks into valuable products, and further studies should be pursued in this direction.

## Conclusions

The transformation of surgical masks into electrocatalysts for oxygen reduction reaction (ORR) and hydrogen evolution reaction (HER) through pyrolysis and functionalization processes is here successfully presented. The electrocatalytic activity towards ORR in neutral and alkaline media shows an increase in the current produced after the functionalization with Fe and Ni. The best‐performing electrocatalyst presented in this work was Mask‐Char‐Fe. Particularly in alkaline media, an onset potential (*E*_on_) of approximately 0.1 V vs. standard hydrogen electrode (SHE) and a half‐wave potential of around −0.02 V vs. SHE were achieved for Mask‐Char‐Fe (0.6 mg cm^−2^ loading). In neutral media, Mask‐Char‐Fe outperformed Mask‐Char‐Ni, which in turn was better than Activated Mask‐Char and Mask‐Char. In neutral media, Mask‐Char‐Fe had an *E*_on_ of approximately 0.3 V vs. SHE and a half‐wave potential of around 0.0 V vs. SHE with catalyst loading of 0.6 mg cm^−2^. Generally, the increase in catalyst loading on the disk improved the kinetics and caused a reduction in the peroxide produced. The electrocatalytic activity towards HER in neutral and alkaline media also shows an increased performance when the char is functionalized with Ni. However, the overpotentials at 10 mA cm^−2^ were quite high and particularly quantified as approximately 0.55 and 0.62 V in neutral and alkaline conditions, respectively. A subproduct of the pyrolysis is the crude oil produced, which is deeply characterized and could be potentially refined, processed, and consequently used as fuel. This work has shown a novel route for transforming waste into valuable resources through pyrolysis processes.

## Experimental Section

### Initial analysis of the surgical mask

Initial characterization of the surgical masks occurred through solid‐state NMR spectroscopy. Data have been acquired using a 9.4 T Avance III Bruker spectrometer using a 4 mm MAS probe. ^1^H spectra were collected with a single‐pulse sequence adopting a π/2 pulse of 2.5 ms and averaging over 128 scans under MAS condition (10 kHz). ^13^C spectra were acquired with ^1^H‐^13^C CPMAS sequence under the same MAS condition. The ^1^H π/2 pulse was 2.5 ms, the delay time 5–200 s depending on the sample, as previously determined with ^1^H experiments, the contact time 2.5 ms, and the signals were averaged over 4–8k scans. High‐power ^1^H decoupling (HPDEC) ^13^C spectra have been collected averaging over 1k scans with a delay time of 50 s and pulse of 4 ms.

### Transformation of the surgical mask into char

Commercially available surgical masks were firstly cut into small pieces using common scissors, followed by crushing into powder using a coffee blender. The obtained powder was placed into a ceramic boat and inserted into a horizontal tubular furnace with a controlled atmosphere (UHP nitrogen at 100 cm^3^ min^−1^). The first pyrolysis process occurred at a heating rate of 5 °C min^−1^ until the temperature of 900 °C was reached. After a dwell time of 1 h, the obtained char was cooled down at the rate of 5 °C min^−1^ and finally collected from the ceramic boat.

### Functionalization of the char

The char obtained from the first pyrolysis was then subject to initial activation and further functionalization in order to: (i) firstly increase the surface area and (ii) create M‐N_*x*_ active sites on the surface. Therefore, obtained char was initially activated by KOH using the methodology reported by Lv et al.^[57]^ Particularly, the char was impregnated with KOH solution (in 15 g ethanol) by adjusting the KOH/char mass ratio at 4. The mixture was then dried in a nitrogen environment at 80 °C under continuous stirring. The obtained sludge was then subjected to heat treatment at 700 °C for 1 h in a tube furnace under the 100 cm^3^ min^−1^ (UHP nitrogen), while the heating and cooling rates were maintained at 5 °C min^−1^. The so‐prepared activated char was subsequently washed with 1 m HCl and millipore water, followed by drying at 80 °C in a vacuum oven for 12 h. After activation, the char was mixed using a mortar with the metal phthalocyanine of interest. For ORR electrocatalyst, the activated char was mixed with 20 wt% of FePc or NiPc. For HER electrocatalyst, the activated char was mixed with 20 wt% of NiPc. After homogenization, the powder was subject to a pyrolysis process in UHP nitrogen at 100 cc min^−1^. The pyrolysis occurred at a heating rate of 5 °C min^−1^ until the temperature of 900 °C was reached, maintained for 1 h, and then cooled down at the cooling rate of 5 °C min^−1^. The black powder was then acid‐washed with boiling 0.5 m H_2_SO_4_ for 15 min to remove oxides and unwanted metallic particles. The material was filtered and washed with deionized water till neutral pH was reached. The electrocatalyst was subject to a second pyrolysis process in a slightly reducing atmosphere (N_2_/H_2_ 95 %:5 %) for an additional hour at 900 °C (heating and cooling rate of 5 °C min^−1^). Lastly, the electrocatalyst was ball‐milled for 2 h with four consecutive cooling breaks of 5 min after every 30 min at the rotation rate of 400 rpm in order to reduce the sample to a fine powder. The sample treatment and acronyms used are reported in Table 1.

### Surface morphology and surface chemistry

The surface morphology and chemistry of the obtained electrocatalyst were studied using several microscopic and spectroscopic tools. SEM (Tescan GAIA 3 equipped with an EDAX Octane Elect EDS, Brno, Czech Republic) was used to identify the texture of the surgical mask and the surface morphology of the electrocatalyst and to perform elemental analysis.

The BET specific surface area was determined by nitrogen adsorption at 77 K using a Micromeritics ASAP 2020 analyzer. The samples were pretreated at 30 μmHg at 473 K for 15 h. The BET surface area was calculated in the pressure range between 0.1 and 0.22 *P*/*P*_0_ while the pore volume was calculated with the BJH method (17.00–3000.00 Å range).

Raman spectroscopy (Labram, Jobin Yvon, France) was used for evaluating structural char features along the synthetic process. Particularly, the G band and D band were identified, after baseline subtraction, and their ratio was used as an indicator of the structural disorder. Raman spectroscopy was employed to evaluate the structural details of the char achieved from the pyrolysis of masks and the subsequent HER and ORR electrocatalysts produced from the obtained char. The Raman spectroscope (LabRam, Jobin Yvon, France) coupled to a microscope (BX40, Olympus, Japan) and equipped with a Long Working Distance 50x objective (Numerical Aperture: 0.60) was used to focus a helium‐neon laser at 632.8 nm for the excitation of the samples under observation. Raman scattering was detected in backscattering configuration using a silicon CCD (Sincerity, Jobin Yvon, France) operating at 200 K as the detector. The recorded spectra were normalized to the maximum intensity of the G band after baseline subtraction. For each sample, the *I*_D_ */I*_G_ ratio and its standard error were evaluated from spectra collected in 10 different points.

### Electrochemical testing

**ORR**: The ORR measurements were carried out using a rotating ring disk electrode (RRDE) technique (Pine WaveVortex RDE system coupled with a Pine bipotentiostat). The ink was prepared by mixing the electrocatalyst (4.5 mg) with 15 μL of 5 % Nafion® D‐520 ionomer solution and 985 μL of isopropanol. The mixture was ultrasonicated for 30 min. A precision pipette was used for depositing the mixture over the glassy carbon of the RRDE and left drying at room temperature. Two catalyst loadings were studied: 0.2 and 0.6 mg cm^−2^. The glassy carbon had a geometric area of 0.2376 cm^2^ and the platinum ring had a geometric area of 0.2356 cm^2^. The experiments were carried out in two different electrolytes: (i) neutral electrolyte composed by 1 m phosphate buffer (pH 7.2), and (ii) alkaline electrolyte composed by a solution of 0.1 m KOH (pH 13). LSV was performed using a three‐electrode configuration using the electrocatalyst over the glassy carbon working electrode, Ag/AgCl 3 m KCl reference electrode, and a platinum wire as the counter electrode. In the case of ORR, the electrolyte was purged vigorously with oxygen for at least 15 min in order to saturate the liquid solution. The potential was scanned from 0.5 to −0.7 V vs. SHE in neutral media and from 0.35 to −0.8 V vs. SHE in alkaline media The electrode had a rotation speed of 1600 rpm. The ring potential was kept at 1.2 V vs. RHE. Disk current (*I*_d_) and ring current (*I*_r_) were recorded along the experiments and used for evaluating the hydrogen peroxide produced (%H_2_O_2_) [Eq. (1)] and the number of electrons transferred (*n*) [Eq. 2]:

(1) $$H_{2}O_{2}\;\left[\%\right]=\;\frac{200\;\times \;\frac{I_{r}}{N}}{I_{d\;}+\;\frac{I_{r}}{N}}$$

(2) $$n=\;\frac{4\;I_{d}}{I_{d\;}+\;\frac{I_{r}}{N}}\;$$

where *N* is the collection efficiency that was 0.38 as reported by the supplier.

**HER**: HER was studied using the rotating disk electrode (RDE) technique and particularly Pine 616 RDE system coupled with Parstat 2273 potentiostat. Ink was prepared by mixing the electrocatalyst (20 mg) in 2‐propanol (2 mL) and then sonicated for 45 min and then drop‐casted with 5 subsequent depositions of 4 μL each (20 μL total) using a precision pipette over the glassy carbon of the RDE and left drying at room temperature. The ink was then covered with 4 μL of 0.05 % Nafion ionomer solution in order to maintain the adhesion of the particles to the glassy carbon support. The glassy carbon had a geometric area of 0.1963 cm^2^. Two electrolytes were used and particularly the neutral electrolyte was composed by 1 m phosphate buffer at pH 7.2, and the alkaline electrolyte was composed by an aqueous solution of 0.1 m KOH. In the case of HER experiments, the electrolyte solution was purged vigorously with nitrogen for at least 15 min in order to remove residual oxygen from the liquid solution. The potential was scanned from 0 to −0.6 V vs. RHE in neutral media and from 0 to −0.9 V vs. RHE in alkaline media.

### Oil synthesis and characterization

During pyrolysis, liquid oil was formed within the tubular chamber. The oil produced at different temperatures (500, 700, and 900 °C) was collected, stored and analyzed using FTIR spectroscopy (Nicolet iS10, Thermo Scientific) equipped with an attenuated total reflectance (ATR) sampling accessory with a diamond crystal (Smart iTR). For each spectrum, 32 scans with a spectral resolution of 2 cm^−1^ were recorded.

^1^H NMR spectra were collected on a solution NMR 400 MHz Bruker Avance spectrometer. From each sample, around 10 mg were dissolved in 750 μl of CDCl_3_ and placed in a 5 mm NMR tube. The ^1^H NMR spectra were obtained with the following acquisition parameters: 90° pulse width 10 ms, spectral width 12 ppm and relaxation delay 2 s. The total number of scans for every experiment was 64 (four dummy scans) and the acquisition time was set at 2.60 s. All ^1^H NMR chemical shifts reported are relative to the peak of CHCl_3_ set at 7.26 ppm.

GC–MS analyses were performed injecting 2 μL of sample (0.1 μg mL^−1^, hexane GC grade) in presence of hexadecane as internal standard into a gas chromatograph (HP 5890 series II)/electron impact mass spectrometer (HP 5972 series) equipped with a HP‐5MS column capillary (30 m length, 0.251 mm inner diameter, 0.25 μm film thickness, 95 % dimethylpolysiloxan). The injection was in a splitless mode with a temperature of 260 °C. The column was eluted at 60 °C for 5 min, followed by a temperature gradient from 60 to 280 °C at 8 °C min^−1^, and 15 min at 280 °C. The gas carrier was helium (99.9999 % purity) at a pressure of 10 psi (1 mL min^−1^). For the identification of condensable species, each peak shown in GC–MS chromatograms was matched with the NIST mass spectral library.

## Conflict of interest

The authors declare no conflict of interest.

1

## Supporting information

As a service to our authors and readers, this journal provides supporting information supplied by the authors. Such materials are peer reviewed and may be re‐organized for online delivery, but are not copy‐edited or typeset. Technical support issues arising from supporting information (other than missing files) should be addressed to the authors.

Supporting Information

## Data Availability

The data that support the findings of this study are available from the corresponding author upon reasonable request.
