# Supplementary material for: Waste Face Surgical Mask Transformation into Crude Oil and Nanostructured Electrocatalysts for Fuel Cells and Electrolyzers
Source: ChemSusChem. 2021 Dec 9;15(2):e202102351. doi: 10.1002/cssc.202102351 (PMC9300040; doi:10.1002/cssc.202102351)
Supplement: Supplementary file 1 — Supporting Information [file CSSC-15-0-s001.pdf]

ChemSusChem

Supporting Information

Waste Face Surgical Mask Transformation into Crude Oil and Nanostructured Electrocatalysts for Fuel Cells and Electrolyzers

Mohsin Muhyuddin, Jonathan Filippi, Luca Zoia, Simone Bonizzoni, Roberto Lorenzi, Enrico Berretti, Laura Capozzoli, Marco Bellini, Chiara Ferrara, Alessandro Lavacchi, and Carlo Santoro*© 2021 The Authors. ChemSusChem published by Wiley-VCH GmbH. This is an open access article under the terms of the Creative Commons Attribution License, which permits use, distribution and reproduction in any medium, provided the original work is properly cited.

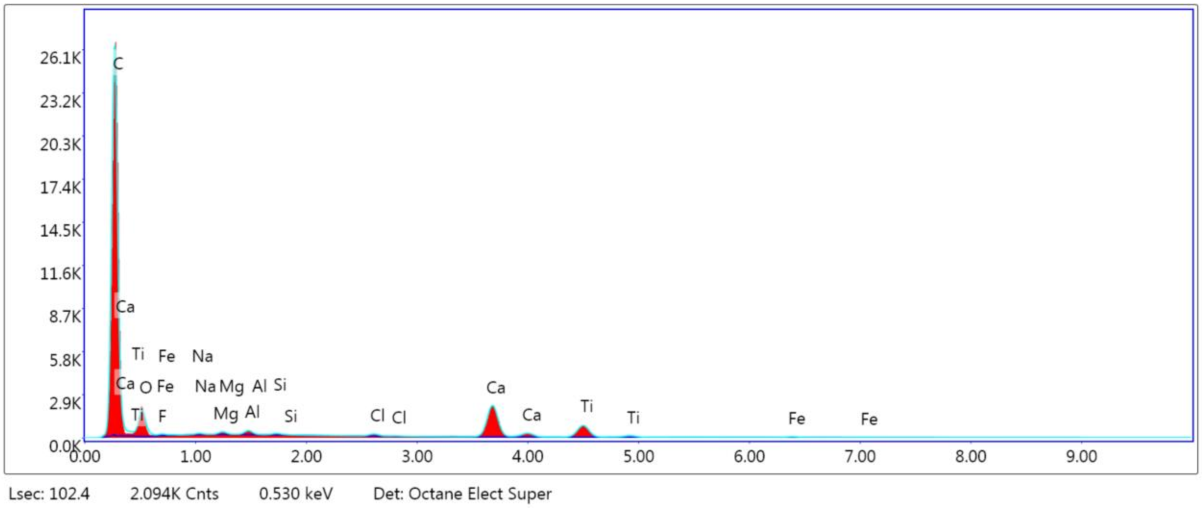

Figure S1. EDS of the Mask-Char

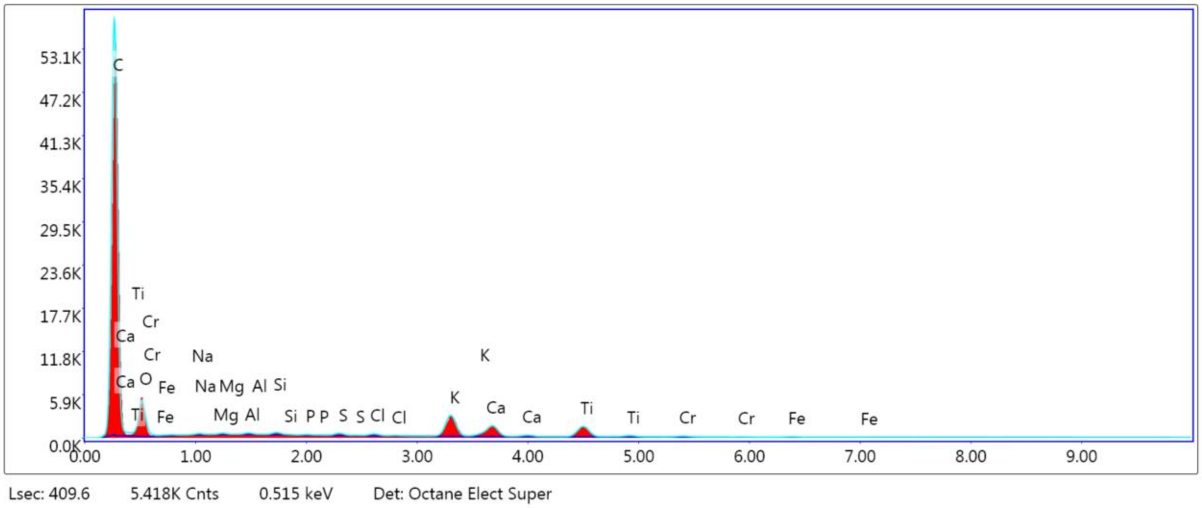

Figure S2. EDS of the Activated Mask-Char

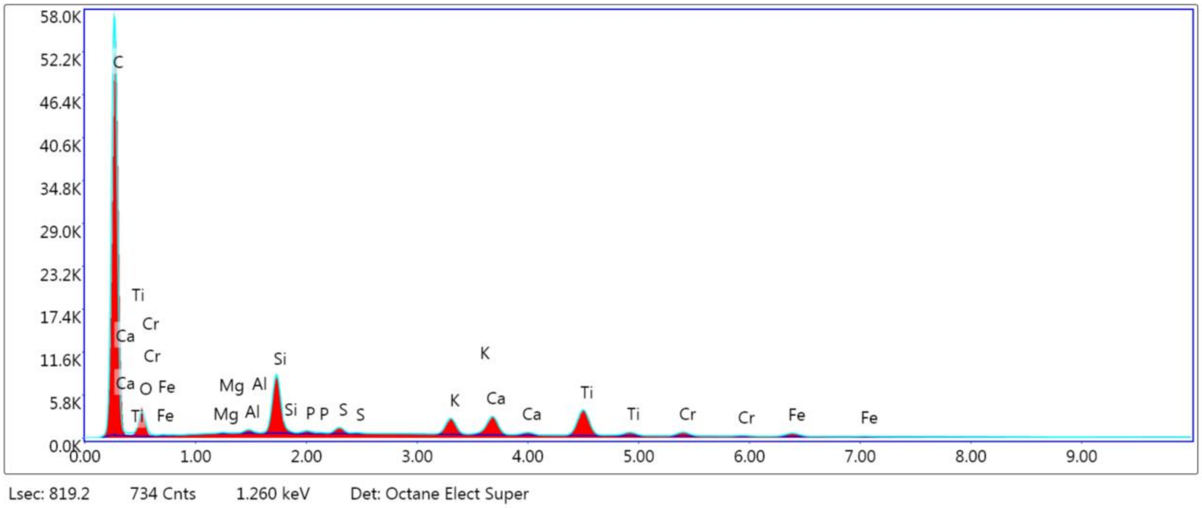

Figure S3. EDS of the Mask-Char-Fe

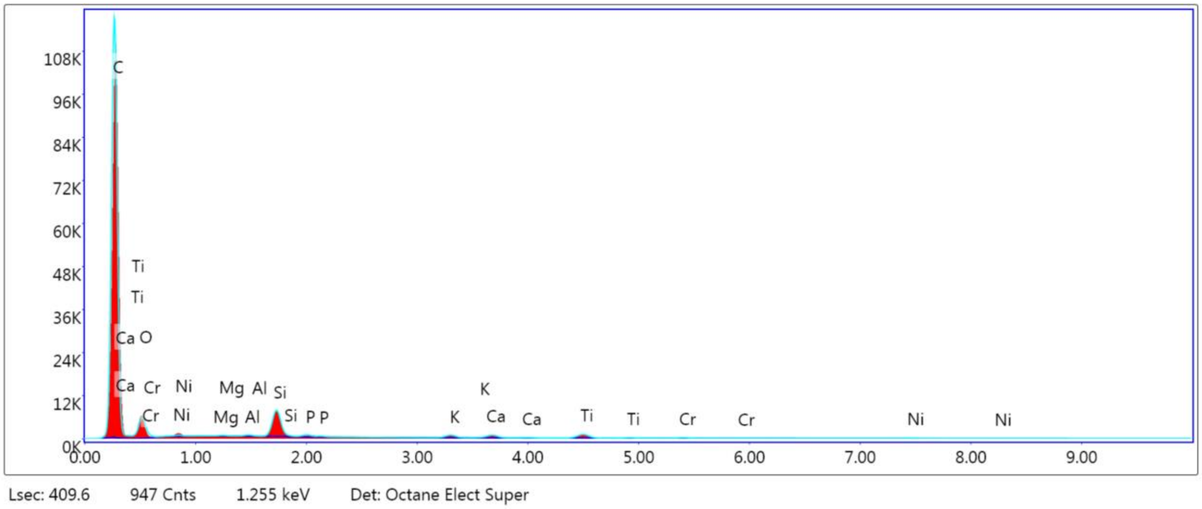

Figure S4. EDS of the Mask-Char-Ni
